# RETRACTION: Expression of Adrenomedullin in Human Colorectal Tumors and Its Role in Cell Growth and Invasion In Vitro and in Xenograft Growth In Vivo

**DOI:** 10.1002/cam4.70172

**Published:** 2024-09-30

## Abstract

**RETRACTION:** E. Nouguerède, C. Berenguer, S. Garcia, B. Bennani, C. Delfino, I. Nanni, L. Dahan, M. Gasmi, J.‐F. Seitz, P.‐M. Martin and L. Ouafik, “Expression of Adrenomedullin in Human Colorectal Tumors and Its Role in Cell Growth and Invasion In vitro and in Xenograft Growth In Vivo,” *Cancer Medicine* 2, no. 2 (2013): 196‐207, https://doi.org/10.1002/cam4.51.

The above article, published online on 29 January 2013 in Wiley Online Library (wileyonlinelibrary.com), has been retracted by agreement between the Journal Editor‐in‐Chief, Stephen Tait; and John Wiley & Sons Ltd. The retraction has been agreed due to duplication of elements found between Figure 2i and 2m and also between Figure 2c, 2g, and 2k. The authors provided a replacement Figure 2 to correct the duplications. However, the editors have lost confidence in the data and conclusions presented in this article as well as the new figure provided.

**RETRACTION:** E. Nouguerède, C. Berenguer, S. Garcia, B. Bennani, C. Delfino, I. Nanni, L. Dahan, M. Gasmi, J.‐F. Seitz, P.‐M. Martin and L. Ouafik, “Expression of Adrenomedullin in Human Colorectal Tumors and Its Role in Cell Growth and Invasion In vitro and in Xenograft Growth In Vivo,” *Cancer Medicine* 2, no. 2 (2013): 196‐207, https://doi.org/10.1002/cam4.51.

The above article, published online on 29 January 2013 in Wiley Online Library (wileyonlinelibrary.com), has been retracted by agreement between the Journal Editor‐in‐Chief, Stephen Tait; and John Wiley & Sons Ltd. The retraction has been agreed due to duplication of elements found between Figure 2i and 2m and also between Figure 2c, 2g, and 2k. The authors provided a replacement Figure 2 to correct the duplications. However, the editors have lost confidence in the data and conclusions presented in this article as well as the new figure provided.

